# Therapeutic treatments for diabetes mellitus-induced liver injury by regulating oxidative stress and inflammation

**DOI:** 10.1186/s42649-023-00089-2

**Published:** 2023-07-10

**Authors:** Chun-Sik Bae, Youngchan Lee, Taeho Ahn

**Affiliations:** College of Veterinary Medicine, Chonnam National University, 77 Yongbong-Ro, Buk-Gu, Gwangju, 61186 Republic of Korea

**Keywords:** Cytochrome P450, Diabetes mellitus, Liver injury, Oxidative stress, Inflammation

## Abstract

Diabetes mellitus (DM) is a metabolic disease that affects all systems in the body, including the liver. Numerous studies have reported that chronic DM etiology and pathogenesis complications implicate oxidative stress, generating reactive oxygen species, such as superoxide anions and free radicals. In addition, pro-inflammatory reactions are also underlying functions closely related to oxidative stress that further exacerbate pathological DM states. The liver is especially susceptible to hyperglycemia-induced oxidative stress and the related inflammation. Thus, anti-oxidation and anti-inflammation therapies are promising strategies for treating liver damage. This review summarizes therapeutic treatments attenuating the generation of oxidative stress and pro-inflammation, which also cause DM-induced liver injury. Although the treatments have several impediments to be solved, these remedies may have clinically important implications under the absence of effective drugs for the damaged liver in DM patients.

## Introduction

Diabetes mellitus (DM) is a worldwide spread and serious metabolic disorder characterized by high blood glucose levels (hyperglycemia). The disease can be classified into two major subclasses: insulin-dependent DM (type 1 DM) and insulin-independent DM (type 2 DM). Type 1 DM, a kind of autoimmune disorder, is attributed to failure of insulin-producing pancreatic beta cells and resulting insulin deficiency, which develops at relatively young ages. In contrast, type 2 DM entails a combination of cellular resistance against insulin and insufficient insulin production, accounting for approximately 90% of the diabetic population (Salsali and Nathan 2006). No distinct evidence has established that type 1 DM and type 2 DM are interconnected, although both DMs share many common symptoms.

DM elicits dysfunction of all systems in the body, including the liver. Several pathways have been studied to induce liver damage in DM patients. Among them, hyperglycemia, mainly caused by insulin resistance and insulin deficiency, affects the metabolisms of lipids, carbohydrates, and proteins and may be the predominant factors inducing liver injuries. In some DM patients, excessive fats, which is closely related to insulin resistance and hyperglycemia, often accumulate in the liver and result in non-alcoholic fatty liver disease (NAFLD). When prolonged without any appropriate treatments, NAFLD can progress to further liver failures such as non-alcoholic steatohepatitis (NASH), cirrhosis, and finally hepatocellular carcinomas (Khandelwal et al. 2021). However, the underlying mechanisms for DM-mediated liver injuries are still unclear and thus further investigation is required to elucidate the molecular effects of DM on tissues in more detail.

Numerous studies have reported to explain the molecular mechanism eliciting DM. Among them, oxidative stress is known to be implicated in the etiology and pathogenesis of chronic complications in DM (Giacco and Brownlee 2010). In addition, inflammatory reactions, which the underlying mechanisms are also closely related to oxidative stress, further exacerbate pathological conditions of DM.

This review describes DM-induced liver injuries and treatments regarding their functional roles and effectiveness against oxidative stress and pro-inflammation, which are also believed to be underlying mechanisms in the development and progression of liver damage in DM conditions. These treatments may be clinically imperative when effective drugs are unavailable to the damaged liver in DM patients.

## Main text

### Oxidative stress and DM

Oxidative stress is defined as an imbalance in the oxidant to antioxidant ratio, generating reactive oxygen species (ROS), such as superoxide anions, hydrogen peroxide, and hydroxyl radicals. Diverse natural biological processes in cells naturally produce ROS as they are necessary for life, such as the bacterial destruction by phagocytes and macrophages and the redox signaling process (Finkel and Holbrook 2000). Aside from these positive functions, oxidative stress plays an essential role in the pathogenesis of various chronic diseases, such as cardiovascular and neurodegenerative diseases, diabetes, and cancer. Excessive production of oxidative stress results in several deleterious events by irreversible modification of biomolecules, including lipids, proteins, and DNA (Forman and Zhang 2021). Regarding diabetes, increased ROS and hyperglycemia damage the pancreatic β-cells and consequently induce type 1 DM.

The ROS are mainly produced by activated Kupffer cells (also known as hepatic macrophages) in the liver through NADPH-oxidase or inducible nitric oxide (NO)-synthase activities (Decker 1990). In this regard, Kupffer cell dysfunction is central to hepatic injuries and contributes to the pathogenesis of NAFLD in DM. However, the liver has elaborate and potent antioxidant systems: superoxide dismutase (SOD), catalase (CAT), and glutathione (GSH)-related enzymes, such as glutathione-*S*-transferase (GST) and glutathione peroxidase (GPX). This defense system scavenges free radicals and hydrolyzes hydrogen peroxides, protecting liver cells from oxidative damage. For example, several studies have proved that decreased SOD and CAT activities, including reduced reduction potentials in a hyperglycemic state, increase oxidative stress and eventually contribute to liver damage. In addition to these enzymes, GSH acts as an endogenous antioxidant in cell viability due to its thiol group oxidizing and producing glutathione disulfide. Moreover, GSH is prominent in the homeostasis of GSH-related enzyme family; reduced GSH levels in diabetic rat livers correlate with decreased activities of GST, GPX, and glutathione reductase activities, including the accumulation of oxidative stress (Han et al. 2006). Therefore, the ratio of GSH to glutathione disulfide in the liver is considered representative of a redox states.

### Inflammation and DM

Substantial evidence corroborates that oxidative stress and inflammation are tightly related, as they interact in various diseases and aggravate pathological conditions (Zuo et al. 2019). For example, the elevated oxidative stress in DM is commonly accompanied by the release of pro-inflammatory cytokines, such as interleukins (IL), tumor necrosis factors (e.g. TNF-α), and prostaglandins produced by Kupffer cells in the liver, which induce apoptosis (cell death) in hepatocytes. Regarding type 1 DM, the combined action of pro-inflammatory cytokines, interferon-gamma, TNF-α, and IL-1β, results in the upregulation of inducible nitric oxide synthase, subsequently elevating NO production (Thomas et al. 2002). Then, NO may induce oxidative stress and apoptosis in several cell types (such as neuronal cells) and participate in host defense, vascular regulation, and neuronal communication (Wei et al. 2000). Several studies verified that IL-6 induces apoptosis in pancreatic islets together with other pro-inflammatory cytokines and is a marker for type 2 DM progression (Tilg and Moschen 2008). TNF-α overproduction also accelerates the inflammation progression and β-cell death in pancreatic islets, eliciting additional insulin resistance in peripheral tissues. Therefore, the regulation and interdependence of oxidative stress and inflammation are crucial in understanding diabetes complications, including DM-induced liver injuries (Li et al. 2016). Furthermore, therapies based on anti-oxidation and anti-inflammation have been attempted as the strategies for treating liver damages from DM and other diseases.

### Cytochrome P450 enzymes and DM

The cytochrome P450 (CYPs) are a superfamily of heme-containing enzymes that functions as monooxygenase. These enzymes metabolize various xenobiotics such as drugs, toxins, carcinogens, and endogenous substrates, including fatty acids and steroids, mainly in the liver. A vital activity of the CYP enzymes is to convert non-polar to polar compounds for phase II enzyme-mediated conjugation in the liver or for direct excretion of the compounds.

Concerning diabetes, many studies have focused on CYP2E1 and CYP1A1 enzymes. CYP2E1, a membrane protein expressed in high levels in the liver, is an ethanol-inducible CYP that metabolizes endogenous (lipid hydroperoxides, ketone bodies, and acetone) and xenobiotic (e.g. ethanol and chlorzoxazone) compounds. In addition, CYP2E1 catalyzes the bioactivation of several procarcinogens and protoxins, including benzene, *N*-nitrosodimethylamine, and *N*-alkylformamides (Surbrook and Olson 1992). CYP2E1 is also involved in ROS production in mitochondria and this capacity is believed to result in lipid peroxidation, which is associated with the etiology and pathology of many diseases, including diabetes (Leung and Nieto 2013). Notably, the catalytic activity and expression of CYP2E1 preponderate in individuals with obesity, NAFLD, and NASH (Aubert et al. 2011). As diabetes is commonly interrelated with the development of NASH, increased CYP2E1expression may elicit liver damage in diabetes by enhancing ROS production. Moreover, it was theorized that CYP2E1-mediated ROS increases oxidative stress and promotes hepatocyte injury, thereby inducing inflammation, hepatic stellate cell activation, and liver fibrosis (Xu et al. 2017).

CYP1A1 is an aryl hydrocarbon hydroxylase that elevates ROS levels in fish and rodent mitochondria and microsomes, potentially resulting from the non-metabolizable ligand such as tetrachlorobiphenyl inhibiting regular catalytic enzyme cycles (Schlezinger et al. 1999). It is also imperative in foreign chemical detoxification and metabolic activation, and an aryl hydrocarbon receptor induces upregulation of CYP1A1 gene (Nebert et al. 2004). Regarding diabetes, while hepatic CYP1A1 and CYP2E1 activity and lipid peroxidation (LPO) were enhanced in STZ-induced diabetic rats, insulin treatment attenuated the enzyme activities and LPO levels (Kuzgun et al. 2020). In addition to oxidative stress, CYP1A1 overexpression also augmented TNF-α and IL-6 production in RAW264.7 cells by enhancing JNK/AP-1 signaling (Tian et al. 2020). These results indicate that CYP1A1 activity and expression levels are closely related to the initiation and progression of diabetes. Similar results with other CYP enzymes, such as CYP1A2 and CYP3A4, corroborate their association with diabetes. In particular, CYP1A2 contributes to intracellular ROS production and the resulting oxidative damage (Nair et al. 2016). However, functional effects of these CYP enzymes on DM remain unclear.

### Management and treatment for DM-induced liver damage

It has been challenging issue to recover DM by safe and effective medications and treatments. Furthermore, DM is a highly complicated disease, exerting hyperglycemia, hyperinsulinemia, hyperlipidemia, inflammation, atherosclerosis, and other detrimental effects. Besides lifestyle changes, such as diet and body weight reduction, specific treatment has yet to be recommended for NAFLD and NASH patients. A combination of conventional anti-diabetic drugs, such as metformin and pioglitazone, and the co-treatment of these drugs with other compounds such as betaine, atorvastatin, and losartan are used to improve insulin sensitivity in the liver (DeFronzo and R. Eldor R, M. Abdul-Ghani, , 2013). However, these treatments may be a clinically subjective issue as they are not radical cures for DM-induced liver injuries.

Current research speculates that oxidative stress is a paramount factor exerting various hepatic lesions in DM patients. As antioxidants may attenuate these harmful effects, oxidative stress regulation is a promising candidate for liver injury treatment. As oxidative stress and inflammation are closely interrelated in the molecular pathogenesis, these strategies can also decrease pro-inflammation and resultant deleterious effects within the body. However, as not all antioxidants will effectively treat liver and other tissue injuries, it should be revealed how antioxidants recover or protect the liver against DM-mediated complications at molecular levels.

### Vitamins

**Fig. 1:**
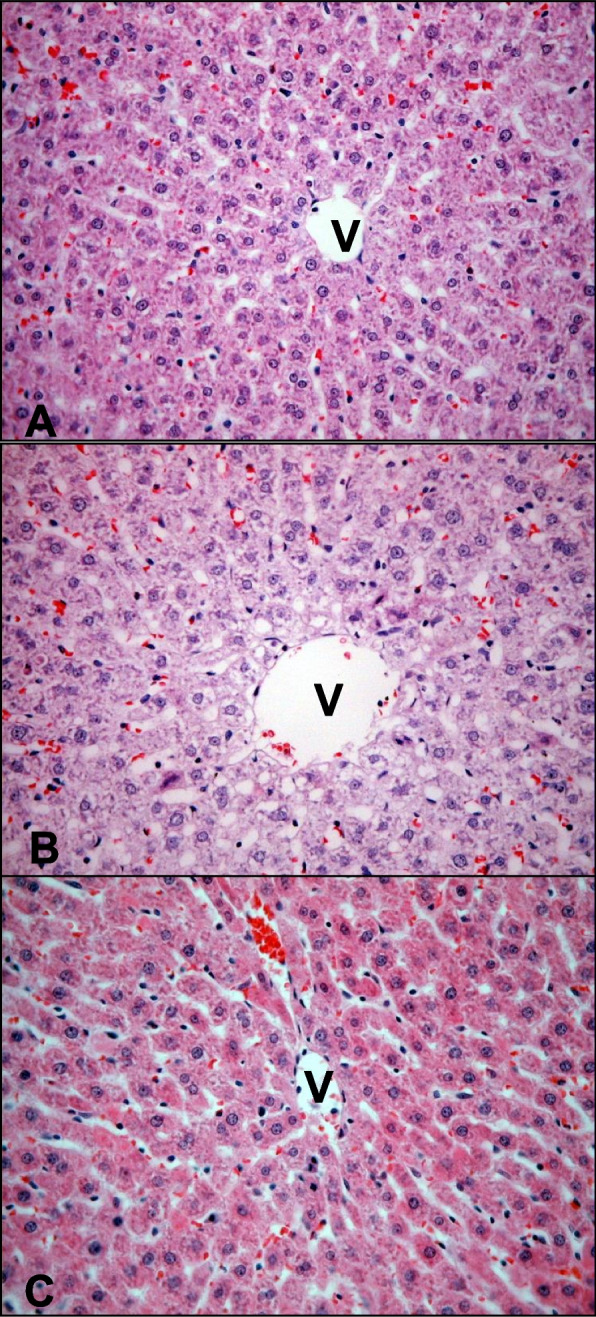
Optical micrographs of the rat liver sections taken four weeks after inducing diabetes and ascorbic acid (AA) supplementation. Micrograph of a rat with (**A**) a control diet, (**B**) streptozotocin-induced diabetes, and (**C**) diabetes and AA supplementation. Arrows indicate microvesicular steatosis. Hematoxylin and eosin staining, 400 ×. Adapted with permission from Ahn et al. 2006

L-ascorbic acid (AA) is a water-soluble vitamin found in various fruits and vegetables. AA has been used as a dietary supplement and an essential nutrient involved in the repair of tissue and diverse enzymatic and immunologic functions in cells. Regarding DM, previous studies demonstrated that AA levels in the plasma and tissues of diabetic patients and animals were lower than in those of normal groups (Will and Byers 1996). In addition, AA supplementation prevents the development of diabetic complications and stabilizes blood vessels (Brownlee et al. 1988). Therefore, reduced AA levels in diabetes are theorized to impair blood vessels and lead to diabetic complications. An earlier study suggested that impairing hepatic regeneration and biosynthesis of AA decreased AA levels in the plasma and tissues of streptozotocin (STZ)-induce diabetic rats (Kashiba et al. 2002). Another study examining the direct relationship between AA and STZ-induced diabetes in rats suggested that AA supplementation is associated with decreased levels of hyperglycemia, hyperlipidemia, and hyperketonemia (Clarke et al. 1996). Moreover, the administration of AA to diabetic rats attenuated an increased CYP2E1 expression and concomitant ROS production and restored histologically modified liver tissue (Fig. 1), confirming the protective effect of AA on the liver injury (Ahn et al. 2006). However, although STZ-treated rats displayed increased expression and activity of hepatic CYP1A, 2B, 2E, and 4A proteins, AA supplementation selectively reduced CYP2E activity and expression (Clarke et al. 1996).

In a study with hypertensive and/or diabetic patients with obesity and high levels of inflammatory markers, AA supplementation significantly reduced the concentrations of C-reactive protein (CRP), IL-6, fasting blood glucose, and triglyceride in blood than the AA-untreated group (Ellulu et al. 2015). Based on these results, the report concluded that AA potentially alleviates inflammation in hypertensive and diabetic obesity patients. A similar study also suggested that co-administration of AA reduces the induction of ER stress and inflammation, including hypoxia, in human pre-adipocytes and adipocytes treated with obesity-associated cell stress (Luo et al. 2022). In spite of these beneficial functions, the molecular effects of AA on inflammatory responses in DM conditions are still unclear, requiring more detailed studies to confirm the anti-inflammatory functions of AA in tissues such as the liver. However, meta-analyses of randomized clinical trials reported that AA supplementation improved glycemic control, cardiovascular risk factors, and oxidative stress in type 2 DM patients (Mason et al. 2021).

Vitamin B12 (or cobalamin) is a micronutrient essential for optimal hemopoietic, neuro-cognitive, and cardiovascular function. Screening diabetic subjects demonstrated that the vitamin deficiency was highly prevalent among type 1 DM and type 2 DM patients (Kibirige and Mwebaze 2013). Thus, DM patients are recommended to receive replacement therapy with either oral or parenteral vitamin B12. However, there are currently no precise supplementation guidelines for patients. In addition to vitamin B12, the concentrations of vitamin A and E, having antioxidant and anti-inflammatory functions, decrease in DM patients, possibly due to controlling excessive production of oxidative stress in glucose metabolism (Valdés-Ramos et al. 2015). In particular, vitamin E reduces the progression of hepatic inflammatory damage, which is notably accelerated by DM-induced oxidative stress (Pazdro and Burgess 2010). The oxygen-scavenging ability of vitamin E inhibits lipid peroxidation and ROS production and further movement to the nucleus, destroying DNA. Earlier studies suggested that vitamin E attenuates oxidative hepatic destruction in various pathological situations caused by ROS formation, although it did not directly reduce the activity and induction of CYP2E1 (Bansal et al. 2005; Chung et al. 2009). Therefore, the supplementations of these vitamins are currently recommended to DM patients. However, it is unknown how effective these vitamins are in restoring DM-mediated liver injury including other tissues. In clinical trials, vitamin E administration reduced fasting plasma glucose and insulin concentrations, including the declines of plasma alanine transferase level and oxidative stress, in overweight people (Manning et al. 2004). These results indicate that vitamin E improves oxidative stress and hepatocellular function.

### Amino acids and proteins

L-cysteine (LC) is a semi-essential amino acid synthesized from methionine in humans, pivotal for detoxifying cellular oxidative stress (Dröge 2005). Several studies have reported that LC lowers oxidative stress and insulin resistance in rats (Blouet et al. 2007). N-acetyl-cysteine supplementation also improves insulin sensitivity in women with polycystic ovaries (Fulghesu et al. 2002). The free sulfhydryl group (the thiol side chain) in cysteine is considered crucial to these biological functions. In addition, LC supplementation can potentially lower the levels of blood glucose, glycated hemoglobin, and oxidative stress in diabetic rat liver (Jain et al. 2009). This study also suggested that cysteine inhibits NFκB and Akt activation, which are apparent in a diabetic liver. NFκB induces TNF-α release, contributing to insulin resistance, and is known as hyperglycemia target (Evans et al. 2005). Oxidative stress also activates NFκB in the liver. Therefore, LC could protect against oxidative stress and be used as a therapy for inflammation and/or inflammation-mediated diseases initiated from a diabetic liver.

In contrast to the beneficial diabetic effects of LC, a large population-based cohort study revealed that nine AAs (phenylalanine, tryptophan, tyrosine, alanine, isoleucine, leucine, valine, aspartate, and glutamate) were significantly associated with decreases in insulin secretion and elevation of fasting or 2-h glucose levels (Vangipurapu et al. 2019). Moreover, five of these AAs (tyrosine, alanine, isoleucine, aspartate, and glutamate) were affiliated with an increased risk of type 2 DM incident. Contrary to these results, however, dietary supplementation of LC and glycine restored GSH synthesis and concentration, and thereby oxidative stress and oxidant damages were lowered in erythrocyte of patients with uncontrolled diabetes (Sekhar et al. 2011). Thus, further studies are needed to investigate the effects of AAs on diabetes at molecular levels.

Albumin, the most abundant circulating protein in the plasma, accounts for approximately 60% of the total plasma proteins. Albumin has several essential physiological and pharmacological functions, including the transport of metals, fatty acids, cholesterol, bile pigments, and drugs. Albumin also acts as a predominant antioxidant in the plasma, a body compartment continuously exposed to oxidative stress (Roche et al. 2008). Previous studies indicated that serum albumin is responsible for more than 70% of the free radical-trapping activity (Bourdon and Blache 2001). Thus, a reduced potential for the scavenging of oxygen free radicals is predicted in patients with hypoalbuminemia. Furthermore, albumin is currently used as a biomarker of systemic oxidative stress, as its redox state shifts to a more oxidized state in response to the severity of pathological condition in various diseases, such as liver and renal failures (Tabata et al. 2021).

More recently, albumin infusion was speculated to attenuate pro-inflammation response, oxidative stress, and consequent liver injury in STZ-induced diabetic rats (Bae and Ahn 2022; Bae et al. 2019); albumin administration reduced the levels of oxidative stress and inflammatory markers, such as IL-6, TNF-α and CRP, which are increased by STZ treatment. In addition, albumin restored liver injury indexes, such as the activities of alanine aminotransferase and aspartate aminotransferase and the levels of triacylglycerol and ketones in the blood. Moreover, albumin also reduced the activity and expression of CYP2E1 enzyme, elevated by STZ in the liver. These results indicate that external albumin infusion alleviates liver damage in type 1 DM. However, whether albumin treatment protects the liver or other tissues in type 2 DM patients or models should be further studied. Concerning this issue, clinical trials suggested that plasma albumin levels are lower in type 2 DM patients than in the normal group, and the decrease is associated with increased adiposity, plasma glucose concentrations, and adipose tissue inflammation, increasing the risk for type 2 DM in humans (Chang et al. 2019). However, the possible effects of albumin treatment on type 2 DM progression or complications have yet to be reported.

In addition to albumin, many studies have considered mealtime whey protein clinically applicable for treating postprandial hyperglycemia in type 2 DM individuals. For example, some studies showed that whey protein slowed gastric emptying and increases insulin secretion and incretin peptides, a group of metabolic hormones that stimulates a decrease in blood glucose levels (Smith et al. 2020). Whey protein is also rich in amino acids that can directly stimulate pancreatic beta cells to secrete insulin, which contributes to the reduction in postprandial glycemia. Thus, whey protein may be benefit in type 2 DM management. However, the optimal dose and timing of whey protein ingestion are yet to be defined, and elaborate studies are required to analyze its long-term effects on overall glycemic control.

### Plant extracts

Active phytochemicals in plants have numerous health-promoting functions and are used as therapeutic agents for various diseases world-wide. Kangen-karyu, a traditional Chinese prescription containing six herb extracts, has been clinically used to treat cardiovascular and cerebrovascular diseases, such as angina pectoris. It has several biological activities, including platelet aggregation inhibition, hypertension suppression, and the recovery of aging-impaired learning and memory. The oral administration of Kangen-karyu to STZ-induced diabetic rats ameliorated hypertriglyceridemia, a STZ-induced detrimental results (Kim et al. 2009). These extracts also reduced lipid peroxidation, an oxidative stress indicator, in serum and hepatic tissue, lowering the expression levels of advanced glycation end-products and their receptor. These results suggest that the extracts may benefit diabetic hepatopathy and DM-induced liver diseases, such as cirrhosis.

Pycnogenol (PYC) is a French maritime pine (*Pinus maritima*) bark extract with high antioxidant activity and can enhance reduction potential, protecting cells from oxidative damage (Packer et al. 1999). In addition, this extract has proven beneficial effects against cancer, diabetes, inflammation, asthma, hypertension, and other diseases (Rohdewald 2002). Regarding diabetes, PYC exhibited antidiabetic effects in a type 2 DM rat model by potentiating the antioxidant defense system (Parveen et al. 2010): the extract supplementation ameliorated thiobarbituric reactive substances, malonaldehyde, and protein carbonyl in type 2 DM rats. It also increased glycogen levels, reduced GSH content, and antioxidant enzyme activities in the diabetic liver. In addition to its anti-oxidative function, PYC also exerts anti-inflammatory effects in tissues, although its molecular actions in the liver are still unknown (Peng et al. 2012). Recently, PYC supplementation clinically improved abdominal obesity, glycemic control, and serum total cholesterol levels in type 2 DM patients, suggesting that PYC may be useful in controlling type 2 DM complications (Navval-Esfahlan et al. 2021). Therefore, these results support the possibility that PYC can be pharmacologically used to restore or protect the liver against diabetes-induced damage.

Due to its anti-oxidative, neuroprotective, cardioprotective, anti-inflammatory, and anti-apoptotic functions, *Ginkgo biloba* (GKB) extract has been also used as pharmacological supplementation to treat ischemic heart disease, hypertension, and arteriosclerosis. These therapeutic functions of GKB may be attributed to induce vasodilation, inhibit atherosclerosis and inflammation development, and repress free radicals (Lu et al. 2007). Regarding DM prevention, GKB is currently believed to contain various active compounds that affect insulin function and production. In studies with type 2 DM patients, GKB extract as an adjuvant exerted beneficial role in improving metformin treatment outcomes, suggesting its potential as an effective dietary supplement for DM control in humans (Aziz et al. 2018). However, despite the wide acceptance of its pharmacological functions, it is still unclear which components in GKB are effective in certain diseases, including diabetes. Moreover, few studies have attempted to reveal the molecular mechanism of GKB in DM patients.

D-pinitol is a widely distributed cycloalkane, a cyclic polyol, and natural compound in the Leguminosae and Pinaceae families. D-pinitol has been pharmacologically evaluated for its effective antioxidant, neuroprotective, hepatoprotective, anti-inflammatory and anti-cancer efficacies (Pandi et al. 2022). Therefore, it has been used to treat diabetes, cancer, and cardiac diseases. Considering DM-induced liver dysfunction, D-pinitol administration attenuated oxidative stress, hyperglycemia, and the ultrastructural alteration of liver, including the increased plasma insulin concentration in STZ-induced diabetic rats (Sivakumar et al. 2010). This study also suggested that D-pinitol decreases the levels of pro-inflammatory markers, such as TNF-α, IL-1β, IL-6, NFκB, and nitric oxide. Thus, it was concluded that D-pinitol protects hepatic tissue from oxidative stress-induced liver damage. Although it is not related to the liver damage, D-pinitol treatment also improved glycemic control and insulin sensitivity in type 2 DM patients (Kim et al. 2012).

**Fig. 2:**
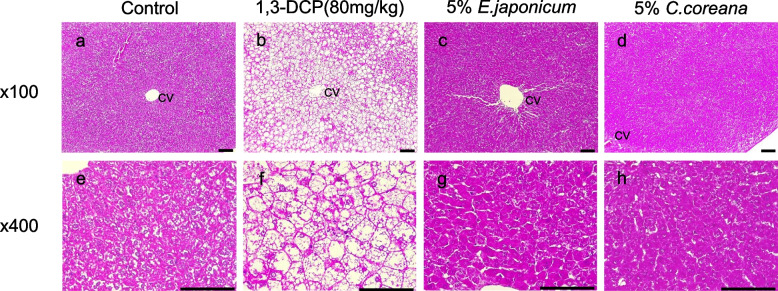
Histopathological features on the protective effects of *Erythronium japonicum* and *Corylopsis coreana* Uyeki against a model of 1,3-dichloro-2-propanol (1,3-DCP)-induced hepatotoxicity. Representative photographs of liver sections of (**a**, **e**) controls indicating glycogen vacuolation and (**b**, **f**) 1,3-DCP (80 mg/kg) exhibiting various histopathological alterations characterized by of hepatocyte degeneration/necrosis around the central vein region, vacuolation, inflammatory cell infiltration, and hemorrhage. Rats treated with 1,3-DCP at dose of (**c**, **g**) 5% *E*. *japonicum*, (**d**, **h**) 5% *C. coreana.* Hematoxylin and eosin staining; Central vein; scale bar = 50 μm (× 100, × 400); 1,3-DCP: 1,3-dichloro-2-propanol. Adapted with permission from Kim et al. 2020

*Erythronium japonicum* and *Corylopsis coreana* Uyeki (Korean winter hazel), which are belong to a perennial herb, contain bioactive compounds such as chlorogenic acid, possessing antioxidant, anti-inflammation and anti-proliferative (consequently anti-cancer) activities (Naveed et al. 2018). Recent studies reported that these plant extracts ameliorated 1,3-dichloro-2-propanol (1,3-DCP)-induced hepatotoxicity in rats, attributed to inhibit CYP2E1 activity and consequently reduce the chemical-mediated oxidative stress (Kim et al. 2020) (Fig. 2). Moreover, this extract treatment restored the 1,3-DCP-mediated decrease in antioxidant enzyme activities, such as SOD and CAT in the rat liver. Histological studies also corroborated the protective effect on the liver. These results are collectively similar to the previous reports regarding functional roles of other plant extracts in diabetes. As Kangen-karyu, PYC, D-pinitol and GKB extracts have proven, the plant extracts are an additional avenue for attenuating liver injuries in DM patients.

### Hormones

Melatonin is the main secretory hormone of the pineal gland in response to darkness, helping circadian rhythms and sleep timing. Additionally, melatonin has free radical-scavenging and antioxidant properties in tissues such as the liver and brain (Taysi et al. 2003). Regarding diabetes, melatonin treatment prevented plasma glucose levels and lipid peroxidation from increasing in STZ-induced diabetic rats (Vural et al. 2001). On the other hand, DM is accompanied by lower serum concentrations of melatonin in diabetic rats (Frese et al. 2009). When analyzed by histochemical methods, melatonin treatment in diabetic rats alleviated STZ-mediated liver damage, which is attributed to its antioxidant properties (Guven et al. 2006). Melatonin is also anticipated to enhance the enzyme activities of anti-oxidative defense system in the liver by removing or attenuating DM-induced oxidative stress. In addition to type 1 DM, prolonged administration of melatonin improved glycemic control in type 2 DM patients (Rezvanfar et al. 2017). Additionally, clinical meta-analyses showed that melatonin significantly reduced fasting blood glucose, insulin resistance, and glycated hemoglobin in type 2 DM patients (Delpino et al. 2021). However, melatonin effect on the liver of type 2 DM should be revealed in molecular levels by further studies. In relation with molecular mechanism of melatonin, hepatic CYP2E1 and ROS levels and resultant liver injury indexes were enhanced in alcohol-fed mice, whereas these deleterious effects of alcohol were significantly alleviated by the treatment with melatonin (Lee et al. 2020). These results confirm that melatonin administration reduces type 1 DM- and alcohol-induced liver injury. However, melatonin is not currently applied or recommended to diabetic patients as dietary supplementation.

There has been some success in employing other hormones, such as estrogen (a menopausal hormone), in treatments to prevent type 2 DM development: menopausal hormone therapy revealed beneficial effects concerning insulin secretion, insulin sensitivity, and glucose effectiveness (Mauvais-Jarvis et al. 2017). However, these hormones are neither approved nor appropriated for preventing type 2 DM due to their complex balance of risks and benefits. Furthermore, more detailed studies are needed to unravel the action mechanisms of the hormones in the body.

### Others

Alpha-lipoic acid (ALA) is a thioctic acid and a sulfur-containing organic compound that is enriched in an animal’s liver, kidney, and heart. It can be also found in whole grains, spinach, cauliflower, and yeast. ALA administration decreased lipid peroxidation and improved antioxidant levels such as SOD activities and GSH content in the liver of methionine-choline deficient diet-induced NAFLD mice (Stanković et al. 2014). In addition, ALA treatment in type 2 DM rats suppressed the activated and expressed protein levels composing inflammasomes (cytosolic multi-protein oligomers in the innate immune system) that stimulate pro-inflammatory responses (Ko et al. 2021). ALA administration also decreased the expression levels of fat synthesis-related proteins and increased those of lipid oxidation-related proteins, suggesting that ALA alleviates NAFLD and hepatic lipid accumulation in the liver. Furthermore, benzene increased CYP2E1 activity and gene expression, resultant oxidative stress, and reduced GSH levels in the rat liver, which these changes were reversed upon ALA administration (El Batsh et al. 2015). These results indicate that ALA is an anti-inflammatory and anti-oxidative reagent and maintains the intracellular antioxidants status conducive to treating diabetes.

Diabetic populations are prone to lipid and lipoprotein abnormalities, which result from insulin resistance. Fatty acids (FAs) affect glucose transporter translocation and insulin receptor signaling, including cell membrane fluidity and permeability. It was thus suggested that FAs have an essential role in insulin resistance and type 2 DM development (Shetty and Kumari 2021). In relation with these functions of FAs, polyunsaturated fatty acids (PUFAs) have attracted attention to attenuate diabetes complications: it decreased oxidative stress, inflammation, and endothelial dysfunction, influence both insulin secretion and insulin resistance, and reduce diabetes risk (Salas-Salvadó et al. 2011). In addition, Omega-3 (n-3) PUFA prevented and reversed high-fat-diet induced adipose tissue inflammation and insulin resistance in mice and its supplementation also protected against beta cell destruction (Baynes et al. 2018). In a clinical trial, n-3 PUFA administration improved insulin sensitivity in Asian type 2 DM patients (Li 2015), although similar results were not found in Western populations. Overall, these results indicate a beneficial effect of PUFAs on diabetes and also potentiate PUFA supplementation for type 2 DM patients. However, studies assessing the beneficial effects of PUFAs on diabetes complications are limited, and some findings conflict with the use of PUFAs for glycemic control.

## Conclusions

**Fig. 3:**
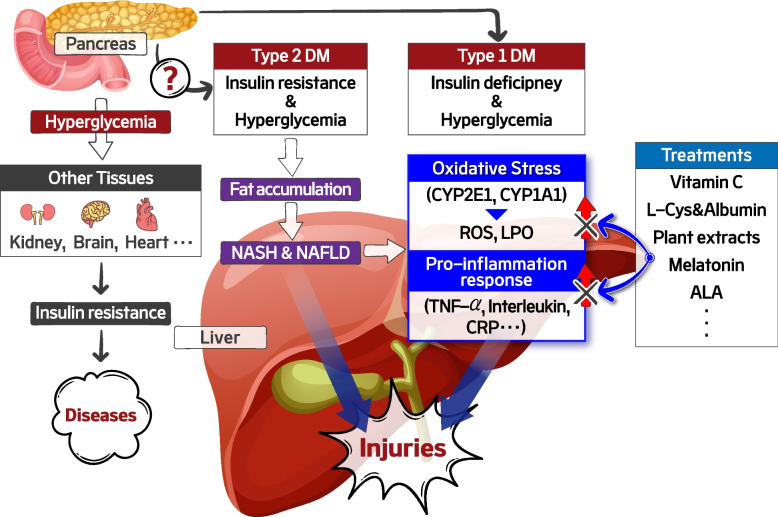
Schematic illustration of DM-mediated liver damage induced by oxidative stress and pro-inflammation response. Oxidative stress and pro-inflammation responses may be main factors contributing to DM-induced liver and other tissue damage. For this reason, anti-oxidation and anti-inflammation therapies can be effective strategies for treating the liver damage

Considering these results collectively, it can be concluded that oxidative stress and related pro-inflammation responses are main factors contributing to DM-induced liver and other tissue damage (Fig. 3). Thus, anti-oxidation and anti-inflammation therapies are promising strategies for treating the liver damage. Furthermore, these treatments are clinically imperative if reliable drugs are unavailable or unable to treat DM-induced liver damage and can also be applied to other diseases with oxidative stress and inflammation as underlying mechanisms. However, these therapeutic remedies still have several impediments, such as further study should be necessary to define the specific ingredients, dosage, and pharmacological effects of these treatments.

## Data Availability

All data generated or analyzed during this study are included in this article, and no datasets were generated or analyzed during the current study. All datasets are available from the corresponding author upon request.

## Abbreviations

DM: Diabetes mellitus
NAFLD: Non-alcoholic fatty liver disease
NASH: Non-alcoholic
ROS: Reactive oxygen species
SOD: Superoxide dismutase
GST: Glutathione-*S*-transferase
CAT: Catalase
GSH: Glutathione
GPX: Glutathione peroxidase
IL: Interleukin
TNF-α: Tumor necrosis factor alpha
CYP: Cytochrome P450
LPO: Lipid peroxidation
AA: Ascorbic acid
STZ: Streptozotocin
PYC: Pycnogenol
GKB: *Ginkgo biloba*
1,3-DCP: 1,3-Dichloro-2-propanol
ALA: Alpha-lipoic acid
